# The efficacy of ophiopogonanone B in treating the cough in mice infected with *Mycoplasma pneumoniae*

**DOI:** 10.3389/fphar.2025.1397543

**Published:** 2025-03-26

**Authors:** Nan-Nan Liu, Bai-Hui Guo, Lei Wang, Xiao-Xi Wang, Xin Wang, Yan-Li Meng, Gui-Xin Tang, Wei-Ming Wang

**Affiliations:** ^1^ College of Traditional Chinese Medicine, Heilongjiang Academy of Chinese Medicine Sciences, Harbin, China; ^2^ Advanced Microscopy and Instrumentation Research Center, Harbin Institute of Technology, Harbin, China; ^3^ School of Chinese Medicine, Southern Medical University, Guangzhou, China

**Keywords:** ophiopogonanone B, cough mechanism, transient receptor potential A1, substance P, calcitonin gene-related peptide

## Abstract

**Introduction:**

Ophiopogonanone B is a potent component of Qinbai Qingfei-concentrated pills (Qinbai), a new traditional Chinese medicine developed by our hospital for the treatment of *Mycoplasma pneumoniae* pneumonia in children. We aim to study how ophiopogonanone B influences the expression of transient receptor potential anchor protein 1 (TRPA1), substance P (SP), and calcitonin gene-related peptide (CGRP) to treat coughing in *MP*-infected mice.

**Methods:**

Ultra-performance liquid chromatography coupled with quadrupole time-of-flight mass spectrometry (UPLC-Q-TOF-MS) was used to detect ophiopogonanone B. Molecular docking of ophiopogonanone B with TRPA1 was performed using Autodock Vina 1.1.2, and subsequent visualization and analysis of docking outcomes were facilitated using Pymol 2.1 and Discovery Studio. For the evaluation of the pathological structure and morphology, lung tissue sections from mice were prepared for animal experiments and subjected to hematoxylin-eosin (HE) and Masson staining. The impact of ophiopogonanone B on the protein and mRNA expression levels of TRPA1, SP, and CGRP in mouse lung tissue was assessed using immunohistochemistry and real-time polymerase chain reaction (RT-PCR).

**Results:**

The samples acquired through Biacore fishing, which were identified and analyzed by UPLC-Q-TOF-MS, confirmed the presence of ophiopogonanone B. This compound exhibited robust and specific binding affinity for TRPA1. Histological staining using HE and Masson techniques revealed that the lung tissue morphology and structure in the ophiopogonanone B-treated group closely mirrored those observed in the blank group. Subsequent immunohistochemistry and RT-PCR revealed a significant reduction (*P* < 0.01 or *P* < 0.05) in the proteins and mRNA expression levels of TRPA1, SP, and CGRP in the lung tissue of mice treated with high and medium doses of ophiopogonanone B.

**Conclusion:**

By decreasing the expression of TRPA1, SP, and CGRP in the lung tissues of mice afflicted with coughing due to *M. pneumoniae* infection, ophiopogonanone B effectively alleviated post-infection cough symptoms.

## 1 Introduction

*Mycoplasma pneumoniae* (MP) is a self-replicating pathogen that lacks a cell wall and occupies an intermediate size range between bacteria and viruses. The primary mode of transmission is the respiratory tract (Rosengarten et al., 2000). MP pneumonia (MPP) is an unconventional form of microbial pathogenic pneumonia initiated by MP infection, characterized by a degree of self-limitation (Kumar, 2018). *Mycoplasma apicalis* can attach to surface receptors located on the ciliated epithelial cells of the respiratory tract, releasing harmful substances that disrupt normal mucociliary clearance and lead to coughing (Parrott et al., 2016). Continuous dry coughing is a common indication of MP infection; however, the effectiveness of typical cough suppressants remains unsatisfactory (Waites et al., 2017). Hence, the quest for medications capable of treating MP, alleviating postinfection cough symptoms, and minimizing adverse effects is of heightened importance.

Traditional Chinese medicine not only has bactericidal and antibacterial effects and eliminates toxins for *Mycoplasma* infections, but also inhibits apoptosis of respiratory epithelial cells, suppresses the systemic inflammatory response, and adjusts the abnormalities of immune function and overall dysfunction (Sun et al., 2020). Qinbai is an inaugural Chinese medicine formulation created by the Heilongjiang Academy of Traditional Chinese Medicine to specifically treat MP in pediatric patients. Qinbai includes the following six ingredients: *Scutellaria baicalensis* Georgi [*Lamiaceae*; *Scutellaria radix*]. *Stemona japonica (Blume)* Miq [*Stemonaceae*; *Stemonae radix*]. *Platycodon grandiflorus* (Jacq.) A. DC. [*Campanulaceae*; *Platycodonis radix*]. *Pheretima aspergillum* (E. Perrier) [*Megascolecidae*; *Pheretima*]. *Ophiopogon japonicus* (Thunb.) Ker Gawl. [*Asparagaceae*; *Ophiopogonis radix*]. *Aster tataricus* L. f. [*Asteraceae*; *Asteris radix et rhizoma*]. Plant names were checked using Medicinal Plant Names Services (http://mpns.kew.org).

Qinbai displays noteworthy efficacy in both the prevention and treatment of MP infections, demonstrating its ability to eliminate MP, while also exhibiting resistance against resorption (Jiang et al., 2014). Xu et al. (2020) demonstrated that Qinbai could reduce the expression of fibrosis factors in MPP, thereby playing an anti-cellulose deposition role (Xu et al., 2020). Belonging to the category of high isoflavones, ophiopogonanone B was the predominant active compound found in Qinbai. Contemporary pharmacological and clinical investigations have revealed that these isoflavones have diverse biological activities and outcomes, including anti-inflammatory, cough-suppressing, antifungal, and vasodilatory properties (Chen et al., 2016).

Transient receptor potential A1 (TRPA1), also known as the ANKTM1 protein, has a substantial stature within the TRP family. It is primarily located in the C-fibers and is widely postulated to exert a pivotal influence on the cough reflex (Grace et al., 2013; De Logu et al., 2016). Activation of TRPA1 triggers a substantial influx of Ca^2+^, culminating in the discharge of neuropeptides, such as substance P (SP) and calcitonin gene-related peptide (CGRP). This cascade results in bronchial constriction and prompts lymphocytes and B cells within the respiratory tract to release inflammatory factors, thereby inducing the onset of coughing (Yang and Li, 2016). Qinbai holds promise as a potential therapeutic agent for post-infectious cough. This is achieved through the inhibition of inflammatory factors, such as TNF-α and IL-1β, suppression of oxidative stress, and augmentation of neuropeptide secretion (Jia et al., 2020). However, to the best of our knowledge, there have been no reported studies on the effects of ophiopogonanone B against MP-induced cough. The objective of this study was to validate the expression patterns of ophiopogonanone B in relation to TRPA1, SP, and CGRP, and to elucidate the mechanism underlying the efficacy of ophiopogonanone B, a vital compound in the management of cough after MP infection. This study provides a theoretical foundation for the clinical application of Qinbai Qingfei-concentrated pills.

## 2 Materials and methods

### 2.1 Chemicals and reagents

Qinbai was supplied by China Harbin Tianheli Pharmaceutical Company (Harbin, China, Lot No. CXZS1000045). The reference standard for Ophiopogonanone B was supplied by Wuhan Tianzhi Biotechnology Company (Lot No. 1316759-83-7, purity: ≥98%). A Rabbit Ultrasensitive Immunohistochemistry Kit (Beijing Zhongshan Jinqiao Biotechnology Company; Lot No. PV-9001), DAB Color Development Kit (Beijing Solarbio Science and Technology Company, lot no. DA1010), and TRIzol Reagent (GLPBIO, United States; Lot No. GK20008), UltraSYBR One-Step Fluorescent PCR Kit (Beijing Kangwei Century Biotechnology Company, Lot No. 29520), TRPA1 Polyclonal antibody (Proteintech, United States; Lot No. 19124-1-AP), Anti -CGRP antibody (Abcam, United Kingdom; Lot No. ab47027), Anti-Substance P antibody (Abcam, United Kingdom; Lot No. ab216414) were used.

### 2.2 Biacore™ test

#### 2.2.1 Pre-enrichment test and ligand coupling test

Based on a previous study (Liang et al., 2022), the Biacore™ (GE Healthcare, United States) Test was used to chemically couple TRPA1 protein to a CM5 chip. A solution containing 200 μL of TRPA1 protein was prepared and injected onto the chip surface under optimal conditions at pH 5.0. The injection was carried out using a volume flow rate of 10 μL/min, and the coupling process lasted for 600 s.

#### 2.2.2 Fishing affinity components using the Biacore™ fishing method

The pre-made Qinbai solution (20 mg/mL) was injected onto the CM5 chip surface using a volume flow rate of 5 μL/min, with a coupling duration of 180 s. A sample of recovery solution (2 μL; 0.5% formic acid solution) was applied to a flow cell and incubated for 20 s to facilitate the dissociation of the binding elements between Qinbai solution and TRPA1, transferring them into the sample recovery solution. Subsequently, the recovered solution, containing binding constituents, was deposited into a 10-μL solution of 50-mmol/L ammonium bicarbonate. This process was repeated for 20 cycles.

### 2.3 Identification of active ingredients using UPLC-Q-TOF-MS

The specimens were dried using a nitrogen blowing apparatus and subsequently reconstituted in a 100 μL methanol solution, followed by centrifugation at 4°C and 12,000 r/min for 20 min. The resulting supernatant was collected and subjected to machine analyses. Chromatographic and mass spectrometric parameters were adopted from the methodology outlined in a previous study (Liang et al., 2022).

### 2.4 Molecular docking simulation experiment

The software Autodock vina 1.1.2, Pymol2.1, and Discovery Studio client were used to simulate and analyze the interaction pattern and binding activity between ophiopogonanone B and TRPA1.

### 2.5 Animal experiments conducted in vivo

#### 2.5.1 Cultivation of MP

MP was revitalized under sterile conditions and propagated in *Mycoplasma* Broth Medium supplemented with 5% fetal bovine serum, at a temperature of 37°C and 5% CO_2_. The mouse nasal instillation model was established after a *Mycoplasma* concentration of 10^6^ CCU.

#### 2.5.2 Experimental animals and *Mycoplasma*

A group of 70 SPF-grade BALB/c mice (six-week-old), with an equal distribution of 35 males and 35 females, was procured from Harbin Medical University. The mice, weighing 20–22 g, all the animals were kept in an isolated room in the SPF maintained at a room temperature of 25°C and humidity level of 60%, with a 12 h light/dark cycle. The animals had access to food and water *ad libitum*. For anesthesia, an intraperitoneal injection of 50 mg/kg pentobarbital was administered. All animals were euthanized using a barbiturate overdose (intravenous injection, 150 mg/kg sodium pentobarbitone) for tissue collection. The animal certificate number for this study is SCXK (Black) 2019-001. MP (American Type Culture Collection; Lot No. ATCC15531) was used. All animal experiments were conducted at the Heilongjiang Academy of Traditional Chinese Medicine. The study protocol was approved by the Protection and Use Committee of the Heilongjiang Academy of Traditional Chinese Medicine (license no. SY3R-2023008).

#### 2.5.3 Development of a mouse model for MPP

Seventy BALB/c mice, evenly split between males and females, were randomly allocated to different groups: blank, model, azithromycin, pentoxyverine, high-dose ophiopogonanone B, medium-dose ophiopogonanone B, and low-dose ophiopogonanone B. Each group consisted of five females and five males. Except for the blank group, mice in each experimental group were anesthetized with ether and then subjected to daily nasal instillation of 20 μL containing 10^6^ CCU of MP for a consecutive period of 3 days. The ophiopogonanone B content in Radix ophiopogonis was obtained from a literature review (Wang, 2014), based on the human dose of Qinbai. Administer different concentrations of the ophingingogonanone B standard solution via gavage to the high, medium, and low dose groups. The high-dose ophiopogonanone B group received a dosage of 44.14 μg/kg/d, the medium-dose ophiopogonanone B group was administered 22.07 μg/kg/d, the low-dose ophiopogonanone B group received 11.04 μg/kg/d, the azithromycin group was given 32 mg/kg/d, and the pentoxyverine citrate group was administered 1.35 mg/kg/d. The blank and model groups were administered equivalent doses of normal saline. After 7 days of continuous administration, a cough-elicitation experiment was performed. Following the cough induction experiment, all animals were euthanized using a barbiturate overdose (intravenous injection of 150 mg/kg sodium pentobarbitone) for tissue collection. Half of the lung tissues were preserved in 10% formaldehyde solution, while the remaining half was frozen at −80°C to facilitate future experiments.

#### 2.5.4 Experiment to elicit cough

Individually, mice from each group were gently placed into an inverted 500 mL glass container containing a cotton ball. Subsequently, 0.5 mL of ammonia water (25% ammonium hydroxide) was drawn using a 1 mL syringe and cautiously dispensed onto the cotton ball (Liang et al., 2022; Chen et al., 2024). The cotton ball was swiftly secured to a beaker. The time span between the initial cough and ammonia addition, known as the cough latency, was meticulously noted and documented. Moreover, the number of coughs occurring within a 3 min timeframe was meticulously observed and recorded. Following cough induction, the mice were humanely euthanized, and their lung tissues were harvested for subsequent investigations.

#### 2.5.5 Hematoxylin-eosin (HE) staining

The lung tissue from each mouse group was sliced, subjected to xylene dewaxing, and stained with HE. Subsequently, the tissue sections were mounted on slides and examined under a light microscope (BX41, Olympus Corporation, Japan) to analyze the histopathological variations among the groups.

#### 2.5.6 Masson’s trichrome staining

The lung tissue sections from each mouse group were dewaxed and hydrated for Masson’s trichrome staining. Following dehydration and clarification, the sections were preserved in neutral resin and examined under a light microscope (BX41, Olympus Corporation, Japan). The collagen volume fraction (CVF) for each group was quantified using Image-Pro Plius software (Media Cybernetics Inc., Bethesda, MD, United States):

$$\text{CVF }\left(\%\right)=\text{collagen area}/\text{total field of view}\times 100\%.$$

#### 2.5.7 Immunohistochemistry

After dewaxing the mouse lung tissue sections and antigen repair, endogenous peroxidase was blocked using hydrogen peroxide. The tissues were incubated overnight at 4°C with primary antibodies against TRPA1, SP, or CGRP: Anti -TRPA1 (1:100), Anti -CGRP (1:500), Anti -Substance P (1:300). The TRPA1 antibody was purchased from Proteintech in the United States, while the Anti-CGRP antibody and Anti-Substance P antibody were purchased from Abcam in the United Kingdom. The secondary antibodies were purchased from Beijing Solarbio Science and Technology Company in China. The reaction enhancer was added dropwise, and add 100 µL or an appropriate amount of the enhanced enzyme-labeled goat anti-rabbit IgG polymer, and incubate at 37°C for 20 min. DAB hatched for 8 min, and counterstained with hematoxylin. The tissues were observed and photographed under a light microscope (BX41, Olympus Corporation, Japan). To assess protein expression (perform a semi-quantitative analysis of the DAB-stained area), the average optical density (AOD) of TRPA1, SP, and CGRP was calculated using Image-Pro Plus v6.0:

AOD=integrated option density/area.

#### 2.5.8 Quantification of mRNA expressions for TRPA1, SP, and CGRP using RT-PCR

Lung tissue samples were extracted from each mouse group, total RNA was isolated using a designated RNA extraction kit, and the RNA concentration of each group was measured using a microplate reader. The mRNA expression levels of TRPA1, CGRP, and SP were detected using the UltraSYBR One Step RT-qPCR Kit. The primer sequences are listed in Table 1.

**TABLE 1:** PCR primer sequence.

Primer sequence	
TRPA1	Forward primer 5′-GCGGTTGGGGACATTGCTGAG-3′ Reverse primer 5′-GGAGCCCTTTGAGCATCTTCT-3′
SP	Forward primer 5′-GAT​CTG​ACC​ATG​CCC​AGC​AT-3′
	Reverse primer 5′-GAA CTG CTG ADD CTT GGG TCT TC-3′
CGRP	Forward primer 5′-GTC​ACT​GCC​CAG​AAG​AGA​TCC​TG-3′
	Reverse primer 5′-CTT​CAC​CAC​ACC​TCC​CGA​CC-3′
β-actin	Forward primer 5′-CTG​GGA​CGA​CAT​GGA​GAA​AA-3′
	Reverse primer 5′-AAG​GAA​GGC​TGG​AAG​AGT​GC-3′

### 2.6 Statistical methods

Data were analyzed using SPSS 22.0. One-way analysis of variance was employed to assess intergroup variations, with the corresponding data presented as mean ± standard deviation (SD). In instances where variance A exhibited significant distinctions among means, an independent samples Tukey’s test was conducted to compare data between the two groups and ascertain notable differences (*P* < 0.05 indicating statistical significance).

## 3 Result

### 3.1 ESI-MS2 data for ophiopogonanone B in the recovered Qinbai sample

**FIGURE 1:**
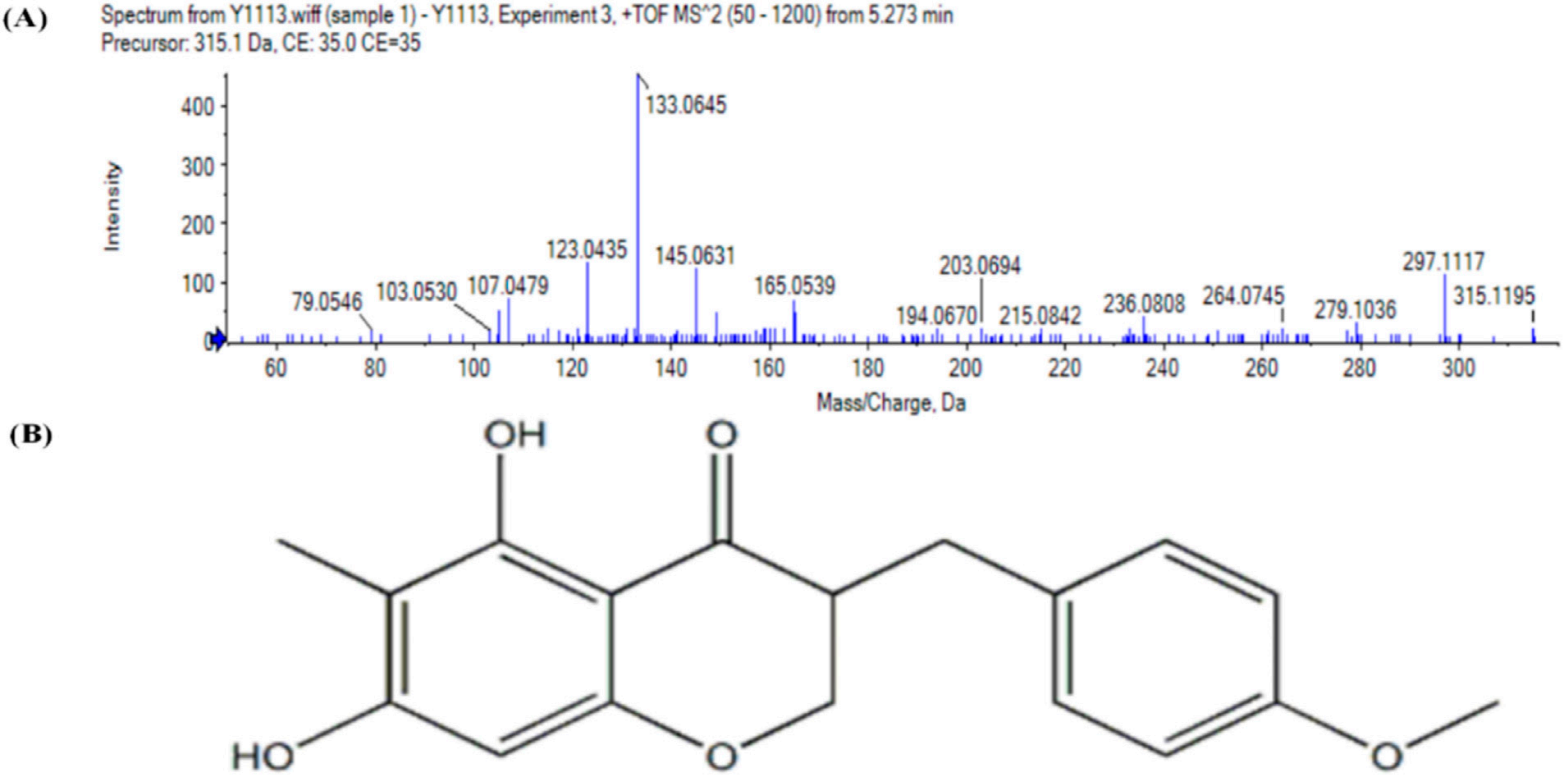
**(A)** Illustrates the analysis of the sample using mass spectrometry, resulting in the acquisition of an ESI-MS2 mass spectrum depicting ophiopogonanone B. **(B)** Ophiopogonanone B Molecular structure formula.

Mass spectrometric analysis revealed the generation of proton ions in the positive ionization mode. Its quasi-ionic peak at m/z 315.122 [M + H] corresponded to [M + H] = 315, and the molecular formula was determined as C_18_H_18_O_5_, with a minor error of −1 × 10^−6^ in the elemental composition analysis. By comparing the principal fragmentation traits of positive ions for ophiopogonanone B, the collected samples were verified as ophiopogonanone B (Figure 1).

### 3.2 Molecular docking results

**FIGURE 2:**
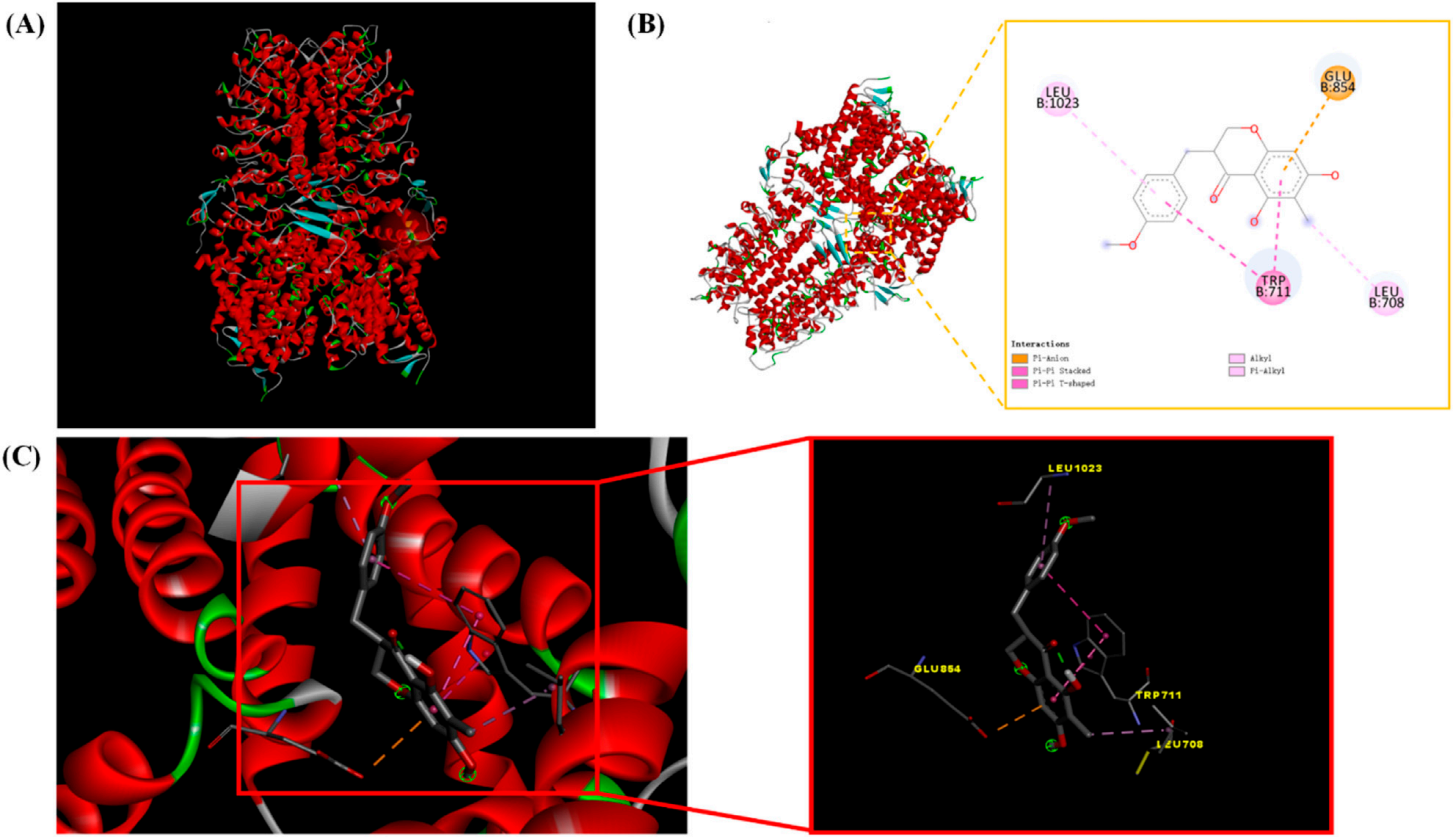
**(A)** Docking active site of ophiopogonanone B with protein TRPA1 molecule. **(B)** Two-dimensional map of docking interactions between ophiopogonanone B and protein TRPA1 molecules. **(C)** Three-dimensional map of the docking interaction between ophiopogonanone B and protein TRPA1 molecules.

Ophiopogonanone B was molecularly docked to the target protein, TRPA1. Molecular docking was performed using Autodock Vina 1.1.2. The receptor and ligand molecules were preprocessed, optimized, and minimized before being input into the software. The docking box was defined to cover the entire protein molecule. For the TRPA1 protein, the docking box coordinates were center_x = 137.928, center_y = −137.536, center_z = 163.585, with a box size of size_x = 126, size_y = 126, size_z = 126. For the mouse TRPA1 protein, the docking box coordinates were center_x = −4.481, center_y = 8.747, center_z = 15.656, with a box size of size_x = 126, size_y = 126, size_z = 126. The molecular docking was carried out using a semi-flexible docking method with 49 runs. A series of complex conformations were obtained and scored. The scoring function of the software is shown in Table 2. The molecular docking results indicate that both ophiopogonanone B and the target protein, TRPA1, were well bound and matched (binding energy is −7.4 kcal/mol). The complexes formed by the docked compounds and proteins were synthesized using Pymol 2.1. The active pocket of TRPA1 was located at the position shown by the red sphere in Figure 2A, and Discovery Studio detected the presence of ophiopogonanone B and TRPA1 according to the binding mode. The amino acid residues that interacted with each other were LEU-708, TRP-711, GLU-854, LEU-1023, and the molecule formed hydrophobic forces with the aforementioned amino acid residues, the molecule matched the protein cavity (Figures 2B, C).

**TABLE 2:** Molecular docking results.

Molecular pair	Binding energy (kcal/mol)	Docking algorithm	Scoring function	Selection criteria for docking sites	Number of docking cycles	Any other relevant parameters
TRPA1- Ophiopogonanone B	−7.4	Semi-flexible docking	$c=\sum_{i<j}f_{ti_{tj}}\left(r_{ij}\right)$	center_x = 137.928 center_y = −137.536 center_z = 163.585	49	size_x = 126 size_y = 126 size_z = 126

### 3.3 Outcomes from the cough elicitation experiment

**FIGURE 3:**
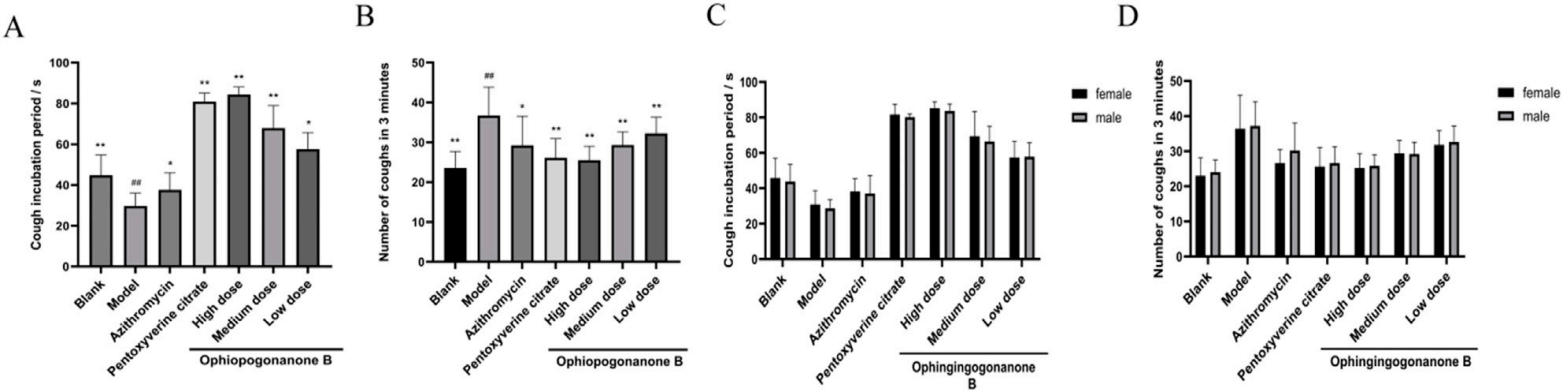
**(A, B)** The effects of ophiopogonanone B on the cough latency and the number of coughs within 3 min in mice. **(C, D)** Comparison of gender differences in cough latency and the number of coughs within 3 min in mice. Data are presented as mean ± SD (*n* = 10). Comparison with blank group, #*P* < 0.05, ##*P* < 0.01; Comparison with model group, *P < 0.05, **P < 0.01.

Ophiopogonanone B was effective in reducing cough frequency in MP infected coughing mice. Compared with the blank group, the model group exhibited decreased cough latency (*P* < 0.01, Figure 3A; Table 3) and an elevated occurrence of cough episodes within a 3 min timeframe (*P* < 0.01, Figure 3B; Table 4), indicating the successful establishment of the model. In contrast to the model group, the azithromycin, pentoxyverine, and ophiopogonanone B groups exhibited prolonged cough latencies (*P* < 0.05, 0.01) and a reduced number of coughs within 3-min (*P* < 0.05, 0.01). Among these groups, a substantial reduction in the number of cough episodes within a 3 min interval was observed in the ophiopogonanone B high dose group, suggesting that the antitussive potency of the ophiopogonanone B high dose group surpassed that of the azithromycin group.

**TABLE 3:** The effects of ophiopogonanone B on the cough latency and the number of coughs within 3 min in mice.

Group	Cough incubation period/s	Number of coughs in 3 min
Blank	44.80 ± 9.40 **	23.50 ± 3.98 **
Model	29.70 ± 5.98 ##	43.50 ± 10.11 ##
Azithromycin	37.60 ± 7.96 *	31.80 ± 7.59 *
Pentoxyverine citrate	80.90 ± 3.94 **	26.10 ± 4.55 **
High dose	84.40 ± 3.56 **	25.50 ± 3.32 **
Medium dose	67.90 ± 10.46 **	29.30 ± 3.13 **
Low dose	57.60 ± 7.66 *	32.20 ± 3.89 **

Data are presented as mean ± SD (*n* = 10). Comparison with blank group, #*P* < 0.05, ##*P* < 0.01; Comparison with model group, *P < 0.05, **P < 0.01.

**TABLE 4:** Comparison of gender differences in cough latency and the number of coughs within 3 min in mice.

Group	Female cough latency period	Male cough latency period	Number of cough by males	Number of coughs by females
Blank	45.80 ± 9.12	43.80 ± 7.90	23.00 ± 4.20	24.00 ± 2.89
Model	30.80 ± 6.41	28.60 ± 4.07	42.40 ± 9.03	44.60 ± 5.59
Azithromycin	38.20 ± 5.84	37.00 ± 8.43	30.40 ± 4.57	33.20 ± 7.82
Pentoxyverine citrate	81.60 ± 4.78	80.20 ± 1.46	25.60 ± 4.42	26.60 ± 3.81
High dose	85.20 ± 3.02	83.60 ± 3.30	25.20 ± 3.39	25.80 ± 2.61
Medium dose	69.40 ± 9.27	66.40 ± 5.18	29.40 ± 2.98	29.20 ± 2.73
Low dose	56.60 ± 5.79	57.40 ± 6.21	31.80 ± 3.34	32.60 ± 3.72

Data are presented as mean ± SD (*n* = 10). Comparison with blank group, #*P* < 0.05, ##*P* < 0.01; Comparison with model group, *P < 0.05, **P < 0.01.

### 3.4 HE staining

**FIGURE 4:**
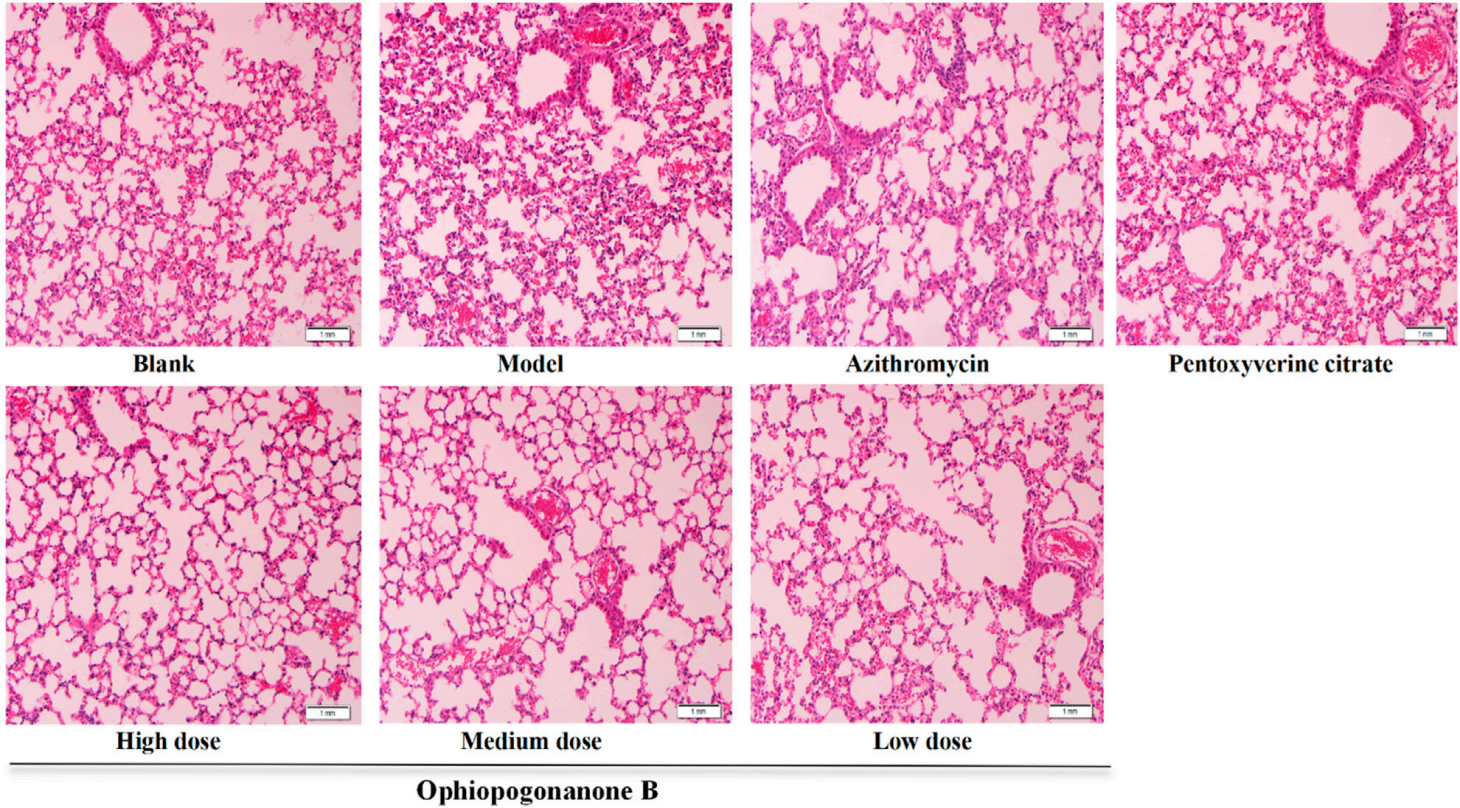
Representative images of HE staining in the lungs of various groups of mice (200× magnifications). (In the model group mice, a significant infiltration of inflammatory cells, thickening of the alveolar walls, and some detached epithelial cells were observed. After treatment, notable improvements were seen in the azithromycin group, pentoxyverine citrate group, and ophiopogonanone B group. The high-dose ophiopogonanone B group showed the most significant improvement).

The lung tissues of mice in the blank group exhibited normal morphology with closely arranged alveolar cells. In the lung tissues of the model group, pronounced thickening of the alveolar wall and frequent alveolar lumen collapse were observed in numerous areas. Moreover, the bronchial epithelium displayed detachment, accompanied by substantial infiltration of inflammatory cells. In the azithromycin group, a discernible enhancement in the lung tissue structure was observed compared to that in the model group. The structural improvements were characterized by greater clarity and a notable reduction in inflammatory cell infiltration. The pentoxyverine group exhibited no substantial improvement in tissue structure, as evidenced by persistent inflammatory cell infiltration. Each ophiopogonanone B dosing group displayed a slight degree of alveolar wall congestion and inflammatory cell infiltration compared with those of the model group. The high-dose group exhibited the most favorable treatment outcomes (Figure 4).

### 3.5 Masson staining

**FIGURE 5:**
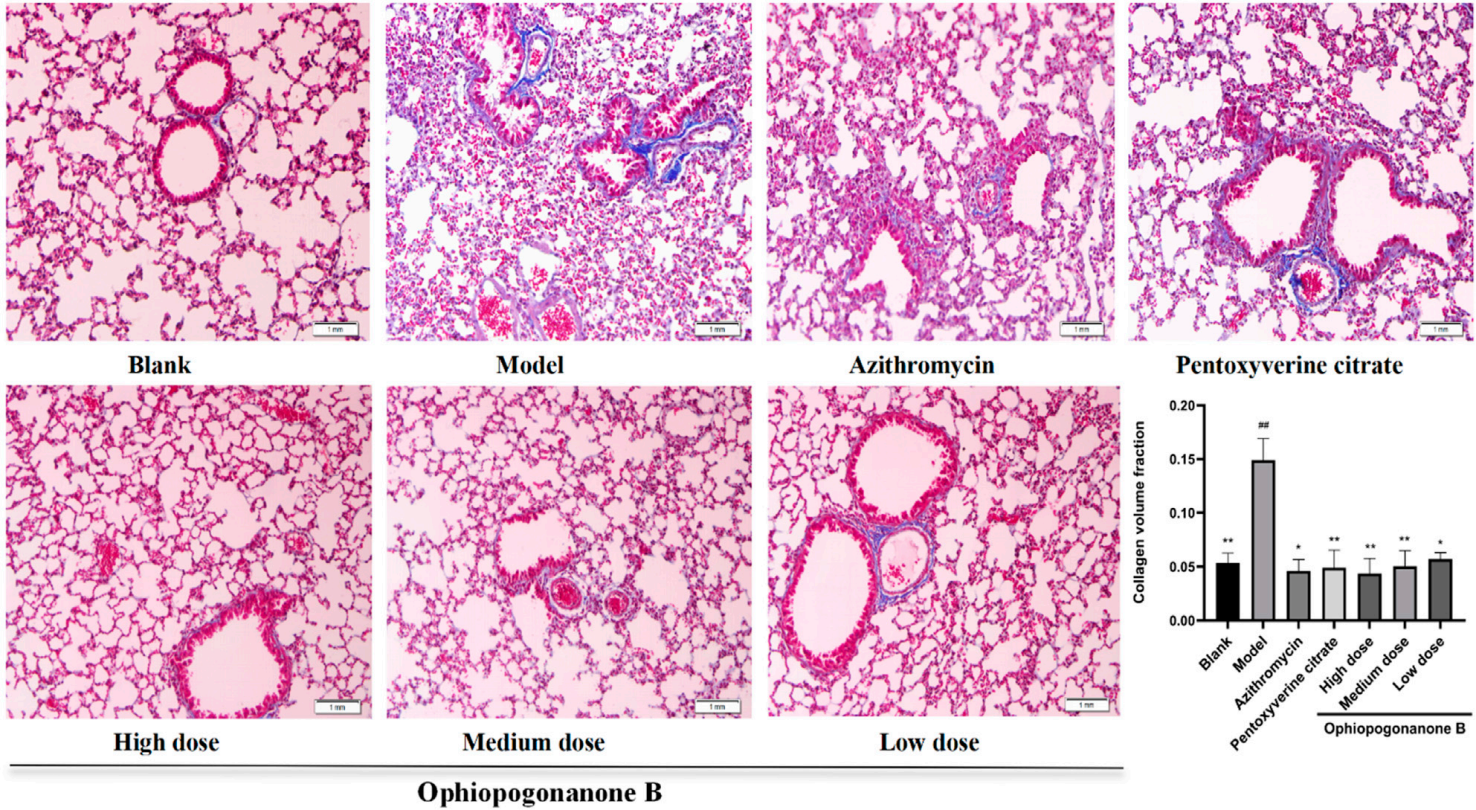
Mouse Masson staining and collagen volume fraction in lung tissue of each group (200× magnifications). Data are presented as mean ± SD (*n* = 10). Comparison with blank group, #*P* < 0.05, ##*P* < 0.01; Comparison with model group, *P < 0.05, **P < 0.01.

Pulmonary tissue sections from the blank group exhibited a closely packed and uniform arrangement accompanied by minimal fibrous deposition surrounding the vascular structures. In the model group, the lung tissue displayed marked disorganization, characterized by the pronounced presence of collagen fibers, alveolar collapse, and substantial aggregation of inflammatory cells along the alveolar walls. In contrast with the model group, the high dose ophiopogonanone B group exhibited a noteworthy reduction in the extent of the blue stained region. The histomorphological characteristics resembled those observed in the lung tissue of the blank group, whereas the efficacy of the treatment decreased with decreasing dosage levels. According to the analysis conducted using Image Pro Plius, the CVF exhibited a marked increase within the model group, displaying a statistically significant difference compared to that of the blank group (*P* < 0.01, Figure 5). In contrast, the ophiopogonanone B group demonstrated a reduction in CVF relative to the model group (*P* < 0.05, 0.01), with the most prominent decrease observed in the ophiopogonanone B high-dose group.

### 3.6 Immunohistochemical assessment of TRPA1, SP, and CGRP protein expression in murine pulmonary tissues across distinct experimental cohorts

**FIGURE 6:**
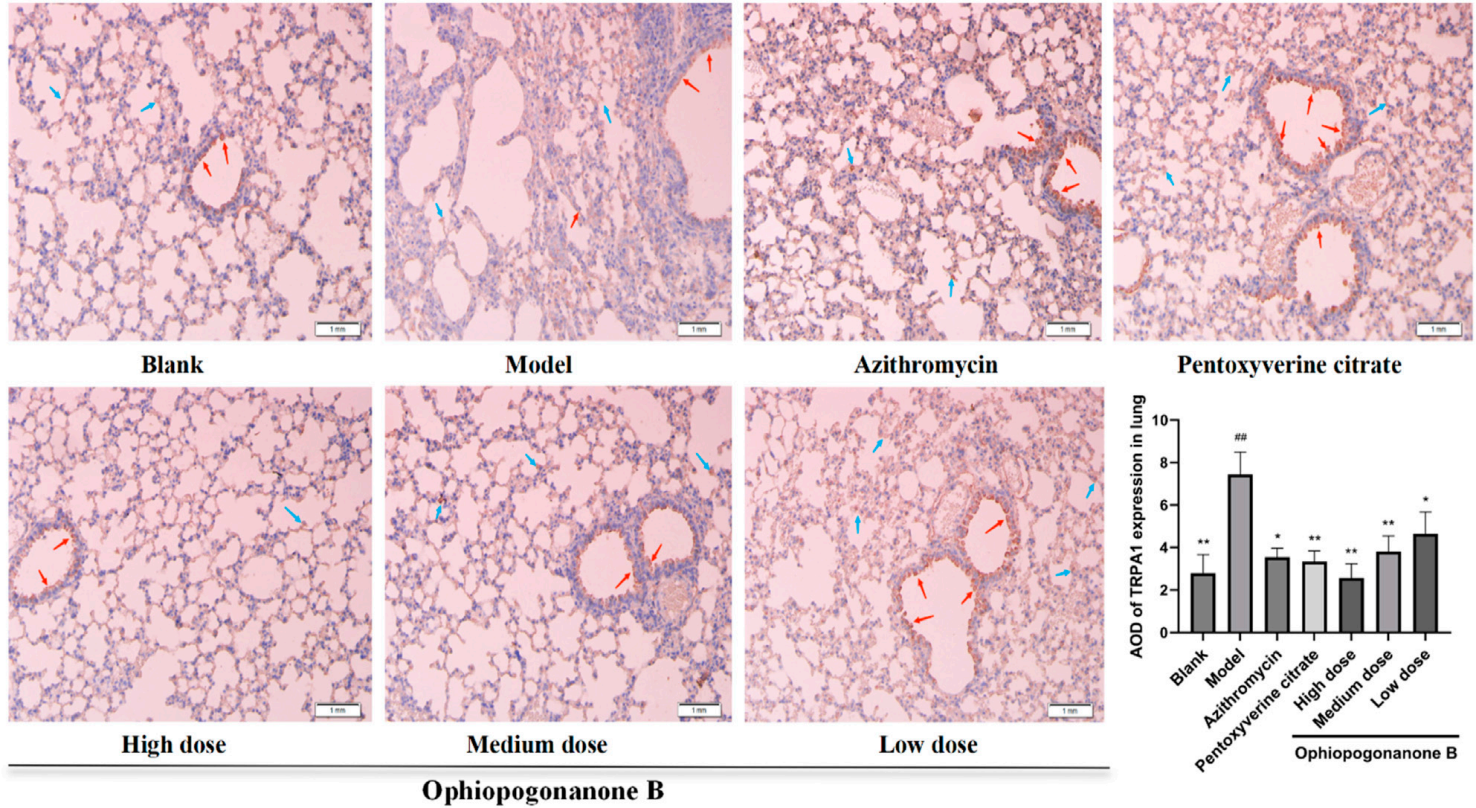
Representative immunohistochemical images and relative quantification of TRPA1 protein expression in mouse lungs (200× magnifications). Data are presented as mean ± SD (*n* = 10). Comparison with blank group, #*P* < 0.05, ##*P* < 0.01; Comparison with model group, *P < 0.05, **P < 0.01. Red arrows represent positive cells in bronchial epithelium, blue arrows represent positive cells in alveolar epithelium.

**FIGURE 7:**
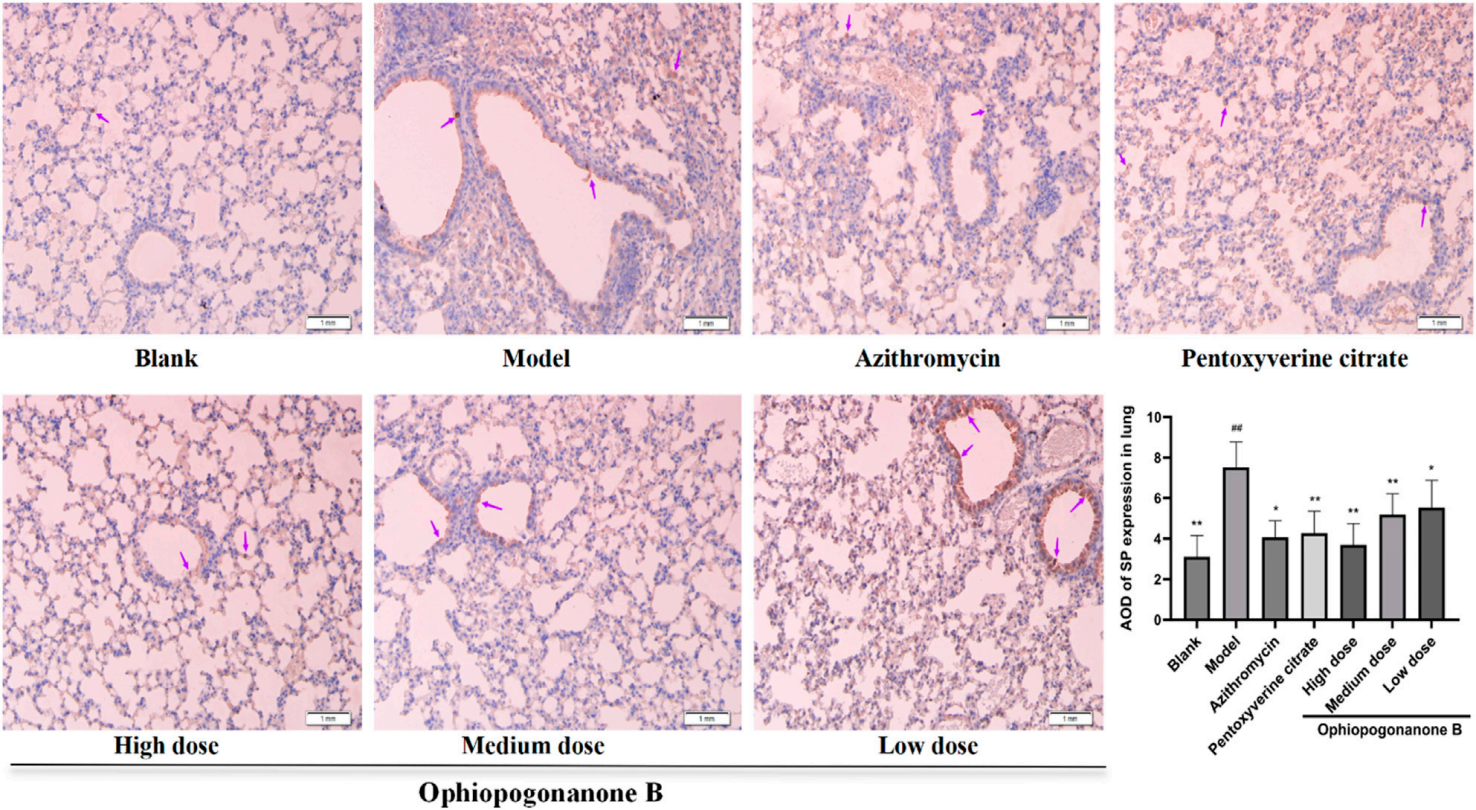
Representative immunohistochemical images and relative quantification of SP protein expression in mouse lungs (200× magnifications). Data are presented as mean ± SD (*n* = 10). Comparison with blank group, #*P* < 0.05, ##*P* < 0.01; Comparison with model group, *P < 0.05, **P < 0.01. purple arrows indicate simply positive cells.

**FIGURE 8:**
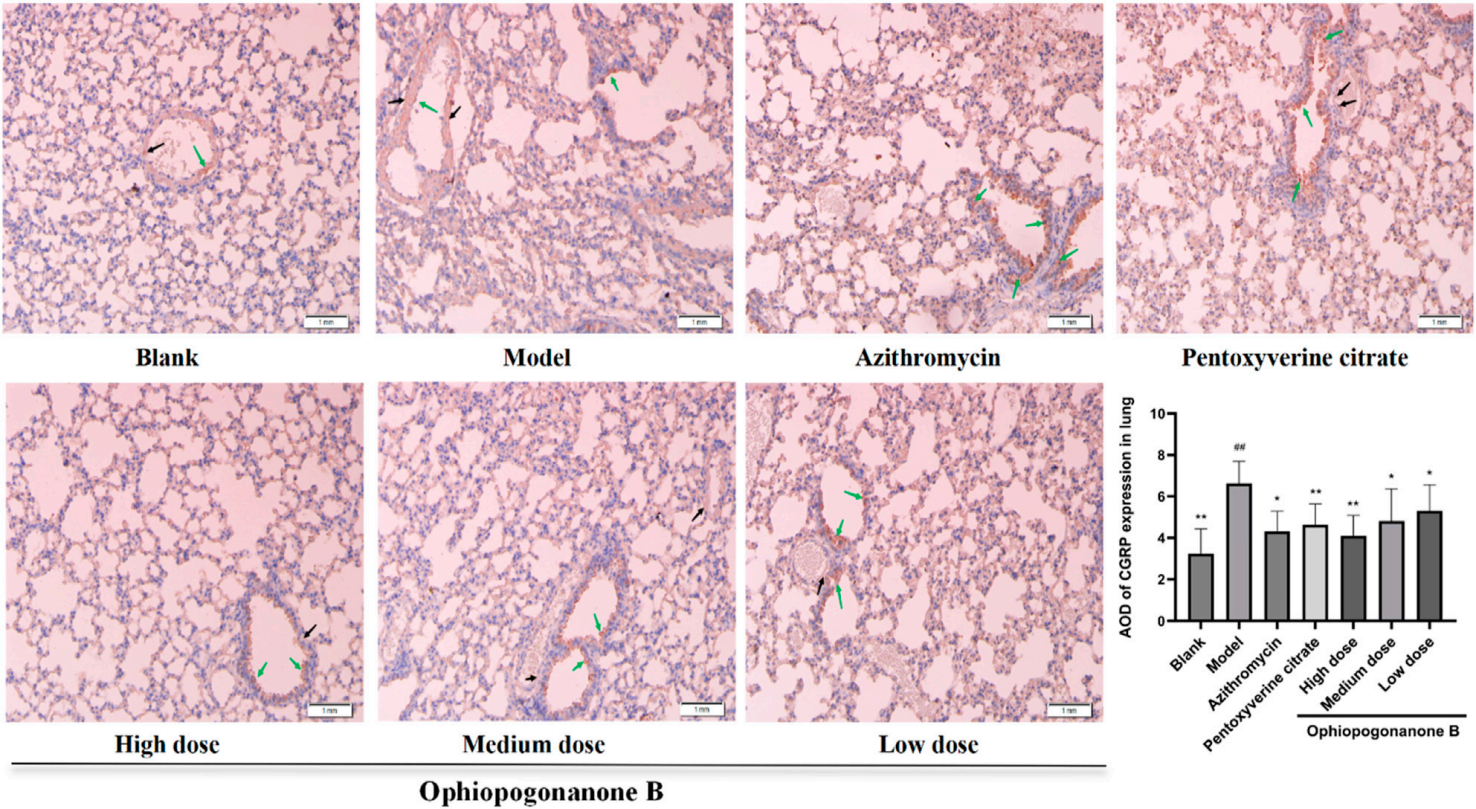
Representative immunohistochemical images and relative quantification of CGRP protein expression in mouse lungs (200× magnifications). Data are presented as mean ± SD (*n* = 10). Comparison with blank group, #*P* < 0.05, ##*P* < 0.01; Comparison with model group, *P < 0.05, **P < 0.01. Black arrows denote positive cells in vascular smooth muscle, green arrows denote positive epithelial cells.

TRPA1, SP, and CGRP levels were significantly higher in the model group than those in the blank group (*P* < 0.01, Figures 6–8). Compared with the model group, the ophiopogonanone B high-dose group showed a significant decrease in TRPA1, SP, and CGRP proteins.

### 3.7 Effects of ophiopogonanone B on *TRPA1*, *SP*, and *CGRP* mRNA expression in lung tissue

**FIGURE 9:**
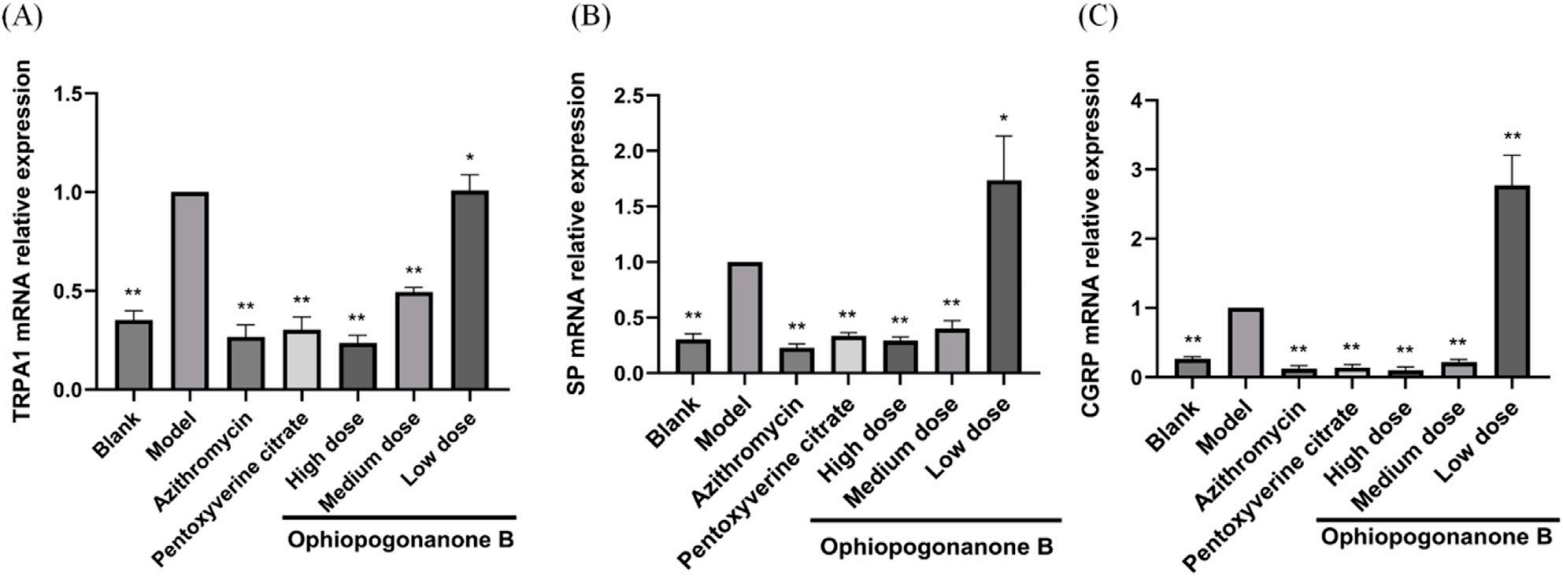
**(A–C)** Impact of ophiopogonanone B on the expression of *TRPA1*, *SP*, and *CGRP* mRNA in murine pulmonary tissue following *Mycoplasma pneumoniae* infection. Data are presented as mean ± SD (*n* = 10). Comparison with model group, *P < 0.05, **P < 0.01.

*TRPA1*, *SP*, and *CGRP* mRNA levels were significantly higher in the model group than those in the control group (*P* < 0.01, Figures 9A–C). Compared with the model group, the Ophiopogonanone B high-dose group showed significantly decreased *TRPA1*, *SP*, and *CGRP* mRNA levels. The efficacy of high dose Ophiopogonanone B was superior when compared with that of azithromycin and pentoxyverine groups.

## 4 Discussion

MP mainly enters the human respiratory tract through airborne droplet transmission, causing damage to the epithelial cells of the respiratory mucosa, slowing down the ciliary activity of the epithelial cells, or even shedding them completely. Additionally, the bronchial wall is hypertrophied, the lumen becomes smaller, and the sputum is not easily discharged, which in turn causes a violent cough (Liu et al., 2022). Qinbai has been shown to be effective in the treatment of MP infection (Yao et al., 2016). Ophiopogonanone B belongs to the category of high isoflavones and is the principal active compound in Qinbai. High concentrations of isoflavonoids exhibit notable antioxidant and anti-inflammatory properties. However, there are few studies on the ophiopogonanone B in the context of post-infectious cough treatment. The current study investigated the underlying mechanisms by which ophiopogonanone B effectively suppressed proteins associated with cough susceptibility, thus providing a therapeutic approach for post MP infection-related cough.

The TRPA1 receptor is not only a “switch” affecting cough, but also an effector closely related to the degree of cough. Moreover, it exhibits robust expression within the sensory nerve C fibers (Undem and Sun, 2020). When MP enters the human lungs through air, TRPA1 transient ion channels may be activated or sensitized, and the activation of TRPA1 initiates an inward flow of calcium ions, thereby fostering the release of inflammatory mediators, including SP, CGRP, and other relevant molecules (Lee et al., 2003). TRPA1 is primarily expressed in sensory nerve endings but is also found in bronchial epithelial cells, bronchial smooth muscle cells, and alveolar macrophages (Ko et al., 2020; Lin et al., 2015). Substance P is a key member of the tachykinin family and is one of the main excitatory neurotransmitters in the non-adrenergic, non-cholinergic (NANC) nervous system of the lung. Nerve fibers containing SP are widely distributed across all levels of the bronchial branches in the lung, occasionally extending into the alveolar walls, and are located in the airway epithelium, the subepithelial basement side, around the airway smooth muscle, and around the submucosal glands and blood vessels (Guo et al., 2010). CGRP is synthesized and stored in C-fibers and epithelial cells, and it is expressed by airway epithelial cells (Pavon-Romero et al., 2021). SP exists together with CGRP in the afferent nerve endings of the C fibers, and the C fibers in the airways are the main source and the main factor triggering the airway neurogenic response (Andrè et al., 2008). SP and CGRP are neuropeptide mediators that can directly activate cough receptors, diminish cough thresholds, and heighten airway receptiveness to a diverse array of stimuli. Consequently, these neuropeptides can directly elicit both acute and chronic cough episodes (Drake et al., 2018). SP augments vascular permeability and exerts potent proinflammatory effects. Concurrently, CGRP facilitates the release of SP, intensifying its biological impact, while also facilitating the transmission of nociceptive signals and inflammatory responses (Russo and Hay, 2023). This suggested that TRPA1 activation triggered the release of inflammatory agents, thereby amplifying the cough reflex. Consequently, suppression of TRPA1 has emerged as a promising therapeutic avenue for addressing post-MP infection-related cough.

In this study, the monomeric component, ophiopogonanone B, was first characterized by UPLC-Q-TOF/MS using a Biacore T200 system. Next, *in vitro* molecular docking was performed to simulate the binding of ophiopogonanone B to the cough protein, TRPA1. The molecular docking plot of ophiopogonanone B with TRPA1 protein showed that ophiopogonanone B bound better to the TRPA1 protein *in vitro*, suggesting its potential role in the anti-MP activity of Qinbai. Surface Plasmon Resonance technology is gaining prominence as a burgeoning research focus in the realm of traditional Chinese medicine-target investigation. Diirone and TGF- β1 proteins specifically bind *in vitro* and inhibit pulmonary fibrosis progression by inhibiting TGF-β1 protein expression (Wang et al., 2019).

Through *in vivo* animal experiments, changes in the expression levels of cough-related factors in the lung tissues of mice after ophiopogonanone B intervention were observed to explore the mechanism of coughing caused by MP. In this experiment, we established a mouse model by nasal instillation of MP bacterial solution using gaseous anesthesia, which offers precise dosage control, rapid onset and recovery, and minimal side effects. Ether is irritating and can cause an increase in respiratory secretions, laryngospasm, and bronchospasm, leading to nonparametric changes that may not accurately reflect true lung function under normal physiological conditions. However, in this experiment, the use of ether was brief, in very small amounts, and there was a considerable time gap before lung function testing, so it had minimal impact on our results. In the future studies, we will try to use other anesthesia methods in order to better conduct experimental studies. Lung tissue slices revealed a high expression of the TRPA1, SP, and CGRP proteins in the model group when ophiopogonanone B intervention had not yet been implemented. Furthermore, microscopic assessment using HE and Masson staining techniques revealed varying degrees of lesions across distinct lung tissue regions. These lesions are characterized by distinct features, including pronounced alveolar wall thickening, conspicuous alveolar lumen collapse, bronchiolar epithelium separation, substantial infiltration of inflammatory cells, and a notable accumulation of collagen fibers surrounding the alveoli. In contrast, MP mice treated with ophiopogonanone B showed good healing of lung tissue morphology and structure, reduced collagen fibers and inflammatory cells in the lung tissue, and significantly reduced expression of TRPA1, SP, and CGRP proteins. These findings confirmed the efficacy of ophiopogonanone B in reducing the release of inflammatory mediators and alleviating subsequent inflammatory responses. Real-time PCR showed that the expression levels of TRPA1, SP, and CGRP in the model group were upregulated, whereas ophiopogonanone B downregulated the expression levels of *TRPA1*, *SP*, and *CGRP* genes.

In summary, ophiopogonanone B exhibited the capacity to diminish cough frequency, ameliorate pulmonary histopathological alterations in mice, mitigate the expression of TRPA1, SP, and CGRP proteins, and effectuate the downregulation of *TRPA1*, *SP*, and *CGRP* mRNA expressions. The results of this study suggest that ophiopogonanone B potentially exerts inhibitory effects on the activation of TRPA1, consequently reducing SP and CGRP release. This action, in turn, contributes to the elevation of the cough threshold, diminished responsiveness of the airways to diverse stimuli, and, ultimately, the amelioration of cough symptoms subsequent to MP infection.

## Data Availability

The original contributions presented in the study are included in the article/Supplementary Material, further inquiries can be directed to the corresponding authors.

## Abbreviations

CGRP, calcitonin gene-related peptide; HE, hematoxylin and eosin; MP, *Mycoplasma pneumoniae*; MPP, *Mycoplasma pneumoniae* pneumonia; Qinbai, Qinbai Qingfei concentrated pellets. SP, substance P; TRPA1, transient receptor potential A1; UPLC-Q-TOF-MS, ultra-performance liquid chromatography coupled with quadrupole time-of-flight mass spectrometry.
