# Supplementary material for: The efficacy of ophiopogonanone B in treating the cough in mice infected with Mycoplasma pneumoniae
Source: Front Pharmacol. 2025 Mar 26;16:1397543. doi: 10.3389/fphar.2025.1397543 (PMC11979145; doi:10.3389/fphar.2025.1397543)
Supplement: Supplementary file 1 [file DataSheet1.docx]

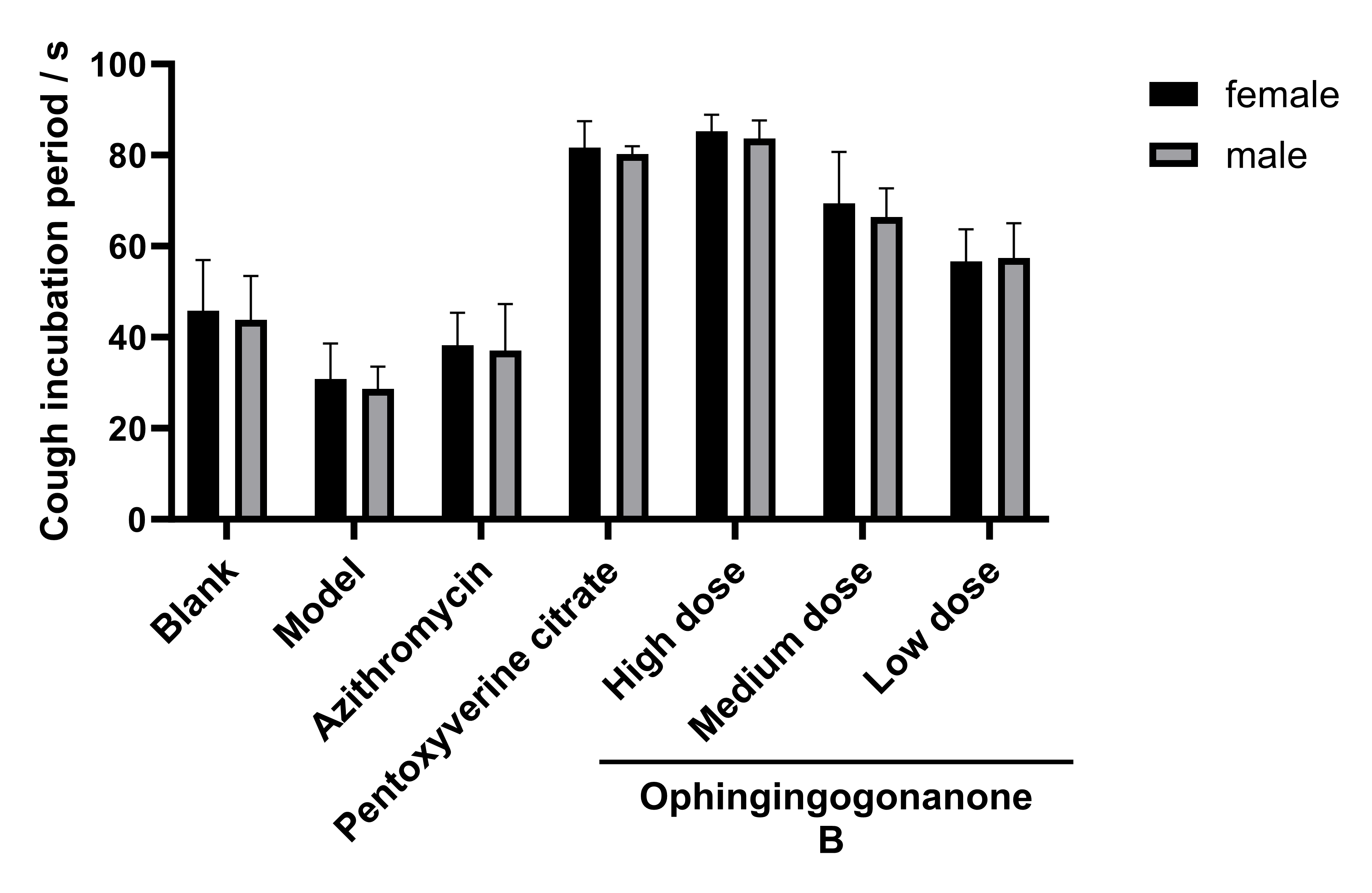

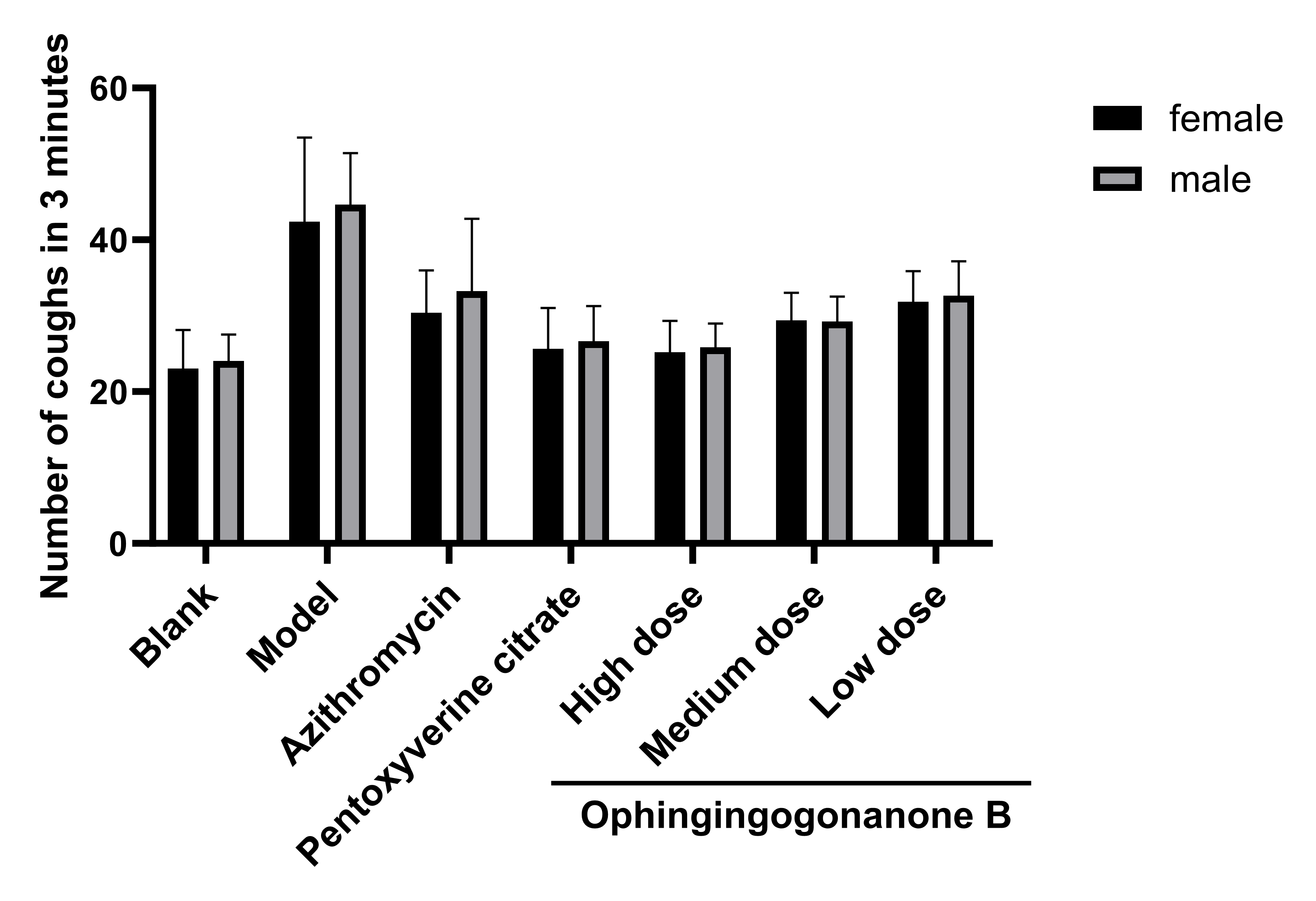


Figure S1. Graph showing the gender differences in cough latency and the number of coughs in mice.

| Group | Female cough latency period | Male cough latency period | Number of cough by males | Number of coughs by females |
| --- | --- | --- | --- | --- |
| Blank | 45.80±9.12 | 43.80±7.90 | 23.00±4.20 | 24.00±2.89 |
| Model | 30.80±6.41 | 28.60±4.07 | 42.40±9.03 | 44.60±5.59 |
| Azithromycin | 38.20±5.84 | 37.00±8.43 | 30.40±4.57 | 33.20±7.82 |
| Pentoxyverine citrate | 81.60±4.78 | 80.20±1.46 | 25.60±4.42 | 26.60±3.81 |
| High dose | 85.20±3.02 | 83.60±3.30 | 25.20±3.39 | 25.80±2.61 |
| Medium dose | 69.40±9.27 | 66.40±5.18 | 29.40±2.98 | 29.20±2.73 |
| Low dose | 56.60±5.79 | 57.40±6.21 | 31.80±3.34 | 32.60±3.72 |

Table S1. Table showing the gender differences in cough latency and the number of coughs in mice.
